# Impact of Hydro-Alcoholic Solvents on the Oil and Phenolics Extraction from Walnut (*Juglans regia* L.) Press-Cake and the Self-Emulsification of Extracts

**DOI:** 10.3390/foods11020186

**Published:** 2022-01-11

**Authors:** Pascale Subra-Paternault, Maria del Pilar Garcia-Mendoza, Raphaëlle Savoire, Christelle Harscoat-Schiavo

**Affiliations:** 1 Institut Chimie et Biologie des Membranes et des Nano-objets (UMR 5248), Institut Polytechnique de Bordeaux, Centre National de la Recherche Scientifique, Université de Bordeaux, Allée Geoffroy Saint-Hilaire, 33600 Pessac, France; p.subra-paternault@cbmn.u-bordeaux.fr (P.S.-P.); pilargm23@hotmail.com (M.d.P.G.-M.); raphaelle.savoire@enscbp.fr (R.S.); 2 School of Basic Sciences, Technology and Engineering, Universidad Nacional Abierta y a Distancia (UNAD), Av. Roosevelt # 36-60, 760042 Cali, Colombia

**Keywords:** walnut press-cake, ethanol, isopropanol, extraction kinetics, phenolic compounds, emulsion

## Abstract

The objective was to evaluate the performance of four hydro-alcoholic solvents to simultaneously extract oil and more polar molecules as phenolics, among others, to produce complex extracts that eventually could self-emulsify after solvent removal. Walnut press-cake was selected as the sourcing material. Extractions were performed as a semi-continuous operation up to a solvent-to-solid ratio of 28, with a fractional collection of the effluent. Among the solvents, labelled by their alcohol content EtOH 58, EtOH 86, iPro 60 and iPro 90 for ethanol (EtOH) and isopropanol (iPro), iPro 90 allowed to reach an oil extraction efficiency of 97% while the recovery for the other solvents was in the range of 30–40%. For both alcohols, the increase of the solvent hydration negatively influenced the oil extraction but positively increased the recovery of phenolics that reached 17.6 mg GAE/g_cake_ when EtOH 58 was used. Several fractions contained enough surface-active material and oil to self-assemble as emulsions. IPro 90 and EtOH 86 showed better performances in the sense that most extracts were able to emulsify, though extraction kinetics pointed out differences. The most hydrated solvents behaved equally, with extraction yields in the same range and a similar but limited emulsifying capacity of only few fractions.

## 1. Introduction

Walnut (*Juglans regia* L.) is categorized as a strategic species for human nutrition by the food and agriculture organization (FAO) and their regular consumption is associated with the reduction of some disease risks, such as cancer and cardiovascular diseases [1]. Walnut oil is used as dressing oil because of its rich nutty flavor and health benefits [2] and as ingredient in cosmetic in dry skin creams, antiwrinkle and anti-aging products [3]. The oil production releases large quantities of partially defatted cakes whose market opportunities are increasingly considered. Though the walnut press-cake composition will depend on cultivars and the oil recovery process, it could be still remarkably rich in oil (18–36%) and proteins (30–45%) [3,4,5]. The residual oil still comprises less than 10% of saturated fatty acids and 62 to 77% of valuable polyunsaturated fatty acids [4,5,6]. Regarding proteins, the four major categories, albumin (water-soluble), prolamin (alcohol soluble), globulin (salt-soluble) and glutelin (soluble in dilute NaOH) are in the range of 7%, 5%, 15–17% and 70–72%, respectively [7,8].

In recent years, much consideration has been given to the secondary metabolites of plants, specially to polyphenols that show antioxidant properties and potent effects in the prevention of diseases associated to oxidative stress [9]. With phenols content in the range of 10–35 mg GAE/g_cake_ [5,10,11,12], walnut press-cake could be harnessed as a cheap and available source of phenolic compounds that have moreover proven their capacity at preserving sensitive oils from oxidation [6,13,14,15]. The phenolic compounds of walnut press cake are mainly hydrolysable tannins and flavonols, the former accounting for 60–80% depending on the nut variety, while the flavonols vary from 26–35% [12,13]. Water, ethanol, methanol and acetone are the most-used solvents for preparing extracts of high antioxidant activities [1,16], but when low toxicity or food applications are targeted, ethanol−water mixtures become the only acceptable solvent. Such mixtures were used to extract phenolics from walnuts [13,17,18].

The oil‘s demand is in constant increase due to nutritional and industrial issues. Large scale facilities of oil recovery are based on a two-step process, involving first a mechanical extraction by pressing followed by a hexane extraction stage to exhaust the press-cake. There is also a range of seeds for which mechanical extraction is not satisfactory, such as hard seeds and oil-poor seeds [19]. Safety, environmental and health concerns have prompted increased interest for alcohols as substitutes to hexane [20,21]. Due to its polarity, ethanol is a weaker solvent than hexane for triglycerides especially in the presence of water, but it can extract a greater variety of polar substances such as phospholipids, soluble sugars and phenolic compounds [22,23]. Isopropyl alcohol has also a high miscibility with oils and is more tolerant to water than ethanol in that the decrease of oil extraction yield with the hydration level of the solvent [24,25,26] or with the matrix moisture [27] is less pronounced. Compared to hexane, its use resulted in higher extraction rates and oil recoveries [27,28], though the reverse was also observed [29]. More polar molecules may be extracted along with the oil such as water, carbohydrates and proteins [26,30].

Oil-in-water emulsions are an integral part of many commercial products used in the food, supplements, personal care, cosmetic, detergent and pharmaceutical industries [31]. Emulsions are thermodynamically unstable systems made of small droplets of one liquid dispersed in another immiscible liquid whose stabilization is achieved by incorporating emulsifiers that adsorb at the interface of the two immiscible fluids. Many of the industrial surfactants are of synthetic origin, but more natural biobased alternatives are increasingly sought after [31,32,33] including in the form of biomass-based particles [34,35]. When used as emulsifiers, extracts are mostly obtained by water extraction in order to recover amphiphilic macromolecules such as proteins and polysaccharides [36,37,38,39,40], though polysaccharides such as saponins can be extracted by ethanol:water mixtures as well [41]. Emulsifying properties of protein hydrolysates or isolates obtained from walnut, specifically, were investigated by several authors [42,43,44]. Another class of surface-active molecules is lecithin, which is a mixture provided with diverse contents and types of phospholipids. Phospholipids can be recovered by water–ethanol mixtures at a yield that increases significantly with the ethanol content [45,46]. Generally speaking, emulsifying properties of extracts obtained by hydrated mixtures or non-aqueous solvents are rarely addressed in literature. Grape seeds tannins, obtained by acetone/water 60:40 *v/v* extraction followed by chromatography for purification, were found to stabilize emulsions [47]. As for non-aqueous solvent, Zhang et al. [48] prepared oil-in-water emulsions using as surfactant a rapeseed extract obtained by supercritical CO_2_. The addition of rapeseed extract at a concentration above 0.4% resulted in an emulsion stability comparable to the addition of 0.2% Tween 20.

The aim of this work was to evaluate the performance of four solvents at producing complex extracts that would contain in their composition oil and polar molecules. By targeting nutritional extracts along with the valorization of a food by-product, we selected walnut press-cake as the source of healthy oil and polyphenols. This work is in the continuity of our previous study in which we focused on phenolic compounds using various methods for their extraction includinga semi-continuous technique with aqueous ethanol as extracting solvent [14]. In the present work, three other hydro-alcoholic solvents were investigated in order to recover at the same time lipophilic and hydrophilic molecules. As a proof of concept, the foreseen property of the extracts is their self-emulsification upon rehydration after the removal of the solvent. Compared to the literature cited above, we therefore intend to produce the oily phase and the emulsifiers by the extraction process itself. Varying the polarity of the solvent is thought to influence the oil and surface-actives content of the extract and thus influence the ability of the extracts to self-emulsify. The extractions were performed in a semi-continuous process that offers the opportunity of fractioning extracts and optimizing solvent-to-solid ratios. The performance of solvents was evaluated on the basis of global extraction yield, oil and polyphenols recovery and the emulsification potential of extracts.

## 2. Materials and Methods

### 2.1. Walnut Cake

The cake was kindly donated by Moulin de la Veyssière, Dordogne, France (www.moulindelaveyssiere.fr, accessed on 5 January 2022). The fruits, of the Lara variety, were grown locally and pressed under mild heating to recover the oil. The cake was stored at −18 °C after its collection. A batch of 100 g was ground and sieved below 600 µm (Retsch, Germany) then stored at −18 °C. The proximate composition of the walnut press-cake was 3% moisture (measured by weight loss during drying at 105 °C), 27% protein (measured by Kjeldahl method using a conversion factor of 5.3), 37% oil (measured by hexane Soxhlet extraction), 5% ash (by incineration at 500 °C for 24 h) and 28% carbohydrates (estimated by mass difference).

### 2.2. Solvents and Reagents

Ethanol (EtOH, 96%) and hexane were purchased from Atlantic Labo (Bruges, France), while 2-propanol (iPro, 99.8%) was from Scharlau (Sharlab, Spain). Deionized water with a resistivity of 15 mΩ·cm (ELGA, Purelab^®^ Option, St. Neots, UK) was used to prepare hydro-alcoholic mixtures and emulsions. The use of ethanol 96 led to slightly different hydration levels for ethanol- and isopropanol-based mixtures. Solvents were labelled in reference to their alcohol content in vol% as iPro 60, iPro 90, EtOH 58 and EtOH 86. Folin reagent, sulfuric acid, anthrone, glucose and gallic acid, were from Sigma-Aldrich (Saint-Quentin-Fallavier, France).

### 2.3. Semi-Continuous Extraction

The semi-continuous extraction consists in flowing the solvent through a bed of walnut cake particles and periodically collecting the effluent [14]. Briefly, five grams of grounded and sieved walnut cake were mixed with glass beads of 2 mm prior being loaded in an empty stainless steel column (19.8 cm × 0.9 cm i.d.) housed in an HPLC oven set at 60 °C. The solvent, heated by a magnetic hotplate stirrer, was delivered at 1 mL/min by an HPLC Waters dual pump. At the column exit, a capillary drove the effluent to a graduated tube immersed in ice that was changed periodically to collect fractions of 7 mL, 7 mL, 7 mL, 22 mL, 44 mL, and 44 mL for an entire extraction time of 140 min. Experiments were randomly triplicated.

### 2.4. Characterization

The collected fractions were transferred to round flasks. An aliquot of 2 mL was sampled for total phenolic quantification while the rest was desolventized under vacuum by a rotary evaporator. The extracted amount in each fraction was quantified by the weight obtained after the solvent removal, taking into account the aliquoted 2 mL. The global extraction yield is reported as cumulative extracted amounts per gram of cake.

The 2 mL aliquots were analyzed for the total phenolic content (TPC) after being vortexed and further filtered on PVDF 0.45 micron or/and centrifuged at 10,000 rpm for 20 min [14]. TPC of extracts was measured by the Folin–Ciocalteu method, using gallic acid as the standard and a calibration curve in a concentration range of 16–350 mg/L. Analyses were performed in duplicate. TPC data are reported in mg of Gallic Acid Equivalents (GAE) per mL of solvent or per gram of cake.

The oil extraction yield, expressed in g oil/g_cake_, was calculated using the mass of oil present in the cake before and after the hydroalcoholic extraction. Masses were obtained by submitting the cake residue to hexane extraction in Soxhlet apparatus (Tecator Soxtec™ -HT2, Hillerød, Denmark) for 4 h. The oil recovery (%) represents the amount of oil extracted by hydro-alcoholic solvents divided by the initial oil amount in the raw cake.

The Soxhlet-defatted cake residues were analyzed for their protein content according to the Kjeldahl method using a conversion factor of 5.3 [49]. Analyses were duplicated.

### 2.5. Emulsification

The collected fractions were evaporated under vacuum to remove the solvent and determine the overall extraction yield. The content of the flask was then recovered by adding 4 or 5 mL of deionized water followed by sonication for 3 min in ultrasonic bath (model Sonorex, Bandelin, Germany, power: 80/240 W). Four mL was the minimum volume to wet the flask walls and recover the extract. The obtained dispersions were observed by optical microscopy (Olympus BX 51 and camera ColorView U-CMAD3, magnification ×10 or ×20, Olympus, Hamburg, Germany). Some dispersions were characterized by laser diffraction using water as the dispersion medium (Mastersizer 2000 equipped with a sample dispersion unit, Malvern Instrument, Orsay, France; He Ne gas laser, beam wavelength of 633 nm). Samples were stored at −18 °C.

### 2.6. Statistical Analysis

Results are presented as mean ± standard deviation calculated from at least two replicates. Statistical evaluation was performed using the R freeware through the Rstudio interface. The significant differences (*p* < 0.05) were analyzed through Tukey’s test.

## 3. Results

### 3.1. Extraction Yields

Extractions were carried out using EtOH58, EtOH86, iPro60 and iPro90 as solvents. Extraction yields obtained at the end of extraction, e.g., at a solvent-to-cake ratio of about 30 mL per g, are reported in Table 1 for the total extracted amount, phenolic compounds and oil.

According to data, the overall extraction capability of the three solvents EtOH 58, EtOH 86 and iPro 60, was similar and in the range of 20 wt%, while iPro 90 exhibited a singular superiority by extracting twice more amounts than other solvents.

For the extraction of phenolic compounds, the extraction capacity of the solvents varied significantly, with a superiority of ethanol versus isopropanol whatever the water content and a better performance of the most hydrated solvents. The solvent of best efficiency for phenols extraction was therefore EtOH 58, while iPro 90 was the worst. Aqueous solvents are effective mixtures to extract phenolics from walnut kernels [1]. The influence of the hydration level was more specifically reported by Fregapane et al. [13], who used EtOH 100, EtOH 80, EtOH 60 and pure water to extract phenolics from pistachio kernels. The extraction rate increased deeply from 5.7 to 16.9 mg GAE/g when the water content varied from 0 to 20% and then slowed down when the water content was further increased to 40% and 100% (18.1–19.8 mg GAE/g). In Odabas and Koca [50], hazelnut skins were extracted by using EtOH 90, EtOH 70 and EtOH 50. The TPC values increased with the solvent hydration from 37 mg GAE/g (EtOH 90) to 59 mg GAE/g (EtOH 50). It is worth noting that not only solubility but phenolics types, their interactions with the matrix and extractions conditions influence the recovery of components.

For oil extraction, there is evidence in the literature that short-chain alcohols such as ethanol and isopropanol could replace hexane in vegetable oils extraction [20,23,25,26,27,51,52], but the presence of water is detrimental to the oil solubility or recovery [30,53,54,55,56]. Our results are consistent with the trends reported in the literature, since a higher hydration level of the extracting solvent decreased the oil extraction yield. The best performance was hence achieved by iPro 90, which allowed the recovery of 98 ± 1% of the oil initially present in the walnut cake, while the recovery for other solvents was of 32 ± 7% for iPro 60 and EtOH 58 and 36 ± 7% for EtOH 87. Comparing now the overall and the oil extraction yields, it can be seen that oil represented a significant part of the global extract. The high extraction yield obtained with iPro 90 was therefore due to its higher capacity for extracting oil.

**Figure 1:**
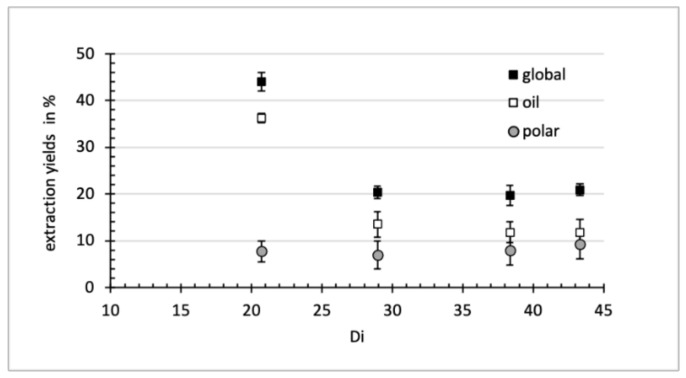
Relation between the various yields (global extraction, oil extraction, polar compounds extraction) and dielectric constant (Di) of the hydro-alcoholic solvents. The yield for polar compounds is calculated as the difference between the global and the oil yields.

Comparing EtOH 100, EtOH 94, iPro 100 and iPro 88 as solvents, Navarro et al. [26] concluded to the best performance of iPro 100 for extracting corn germ-bran oil while ethanol and aqueous mixtures were better for the extraction of carbohydrates. To better appraise the difference of polarity, several authors [25,26,51] correlated the extraction capacity of solvents to their dielectric constant, which was considered as being a key parameter in determining solute–solvent interactions. Dielectric constants of mixtures were calculated (Table 1) and correlated to the various yields (Figure 1). Values were calculated using the equation proposed by Tir et al. [51]*,* Di = Di_A_w_A_ + Di_w_w_w_, where w_A_ and w_w_ are mass fractions of alcohol (A) and water (W), respectively, and Di_A_ and Di_w_ are dielectric constants at 60 °C of pure alcohols [26] and pure water [57], respectively.

The mixing of alcohols with water increased the dielectric constant of the prepared mixtures. The solvent of the lowest dielectric constant, isopropanol, exhibited the best performance for oil extraction, whereas solvents of higher Di (EtOH 86, EtOH 58, iPro 60) led to worse performances with no significant differences between them. In comparison, the yield of polar compounds extraction, evaluated by the difference between the global and the oil extraction yields, ranged between 7 ± 3% and 9 ± 3% but the difference was not statistically significant. By increasing the hydration level of solvents, the solvent increases its ability to extract non-oil compounds [21] such as carbohydrates [26] and proteins [27,54]. In the cited references, the trends were evaluated for ethanol- or isopropanol-based mixtures with a water content varying from 0 to 12wt%. It can be inferred that the 10–14% water content used in this work is sufficient to extract the majority of polar species, and that increasing this proportion beyond 10–14% has no additional impact.

**Figure 2:**
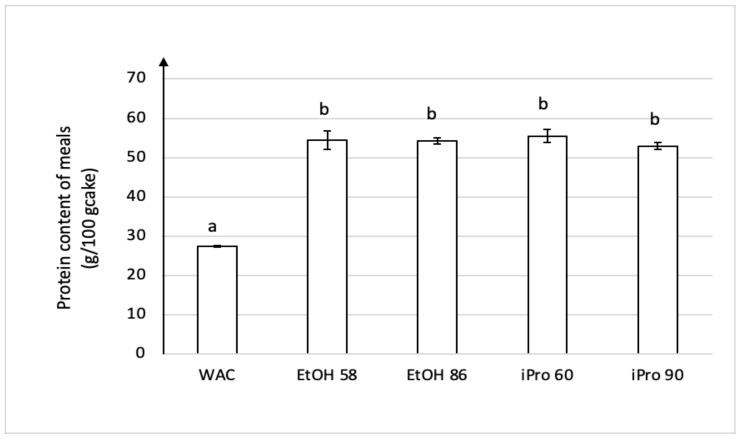
Protein content of meals obtained after extraction with hydro-alcoholic solvents followed by Soxhlet defatting with hexane. WAC label corresponds to the untreated walnut cake. ^ab^ Different letters mean significantly different values at *p* < 0.05.

The protein content of defatted cake residues obtained from extractions with hydro-alcoholic solvents followed by Soxhlet extraction by hexane was evaluated (Figure 2).

Due to the sequence of extractions, the protein content in the treated meals was higher than the protein content of the raw cake (27.4 ± 0.2 g/100 g_cake_). Statistics moreover confirmed that there was no significant effect of the extracting solvent on the cake residual protein content. This is consistent with the previous comment on the polar compounds extraction yield stating that a 10% water content in the extracting solvent was sufficient to extract most of the polar substances. In fact, proteins of the walnut kernel are mainly composed of glutelins (about 70% of the kernel proteins) and globulins (about 18%) that are soluble in dilute NaOH and saline media, respectively [8]. In the cited study*,* the extraction of walnut flour by EtOH 70 led to a low yield of about 3% because of the presence in small proportion only of the alcohol-aqueous-soluble prolamins [8,18]. The effect of hydro-alcoholic extraction on the treated meal proteins was evaluated in literature for sesame cake [24], corn germ-bran pellets [26] and rice bran pellets [25] using absolute ethanol, EtOH at 6 wt% of water, absolute isopropanol and iPro at 12 wt% of water. At 60 °C, only the cakes treated by absolute iPro showed a slightly higher protein content or nitrogen solubility index for corn germ- or rice bran-pellets, respectively, whereas for sesame, significant differences were not observed among the NSI values.

### 3.2. Extraction Kinetics and Aspect of Collected Fractions

**Figure 3:**
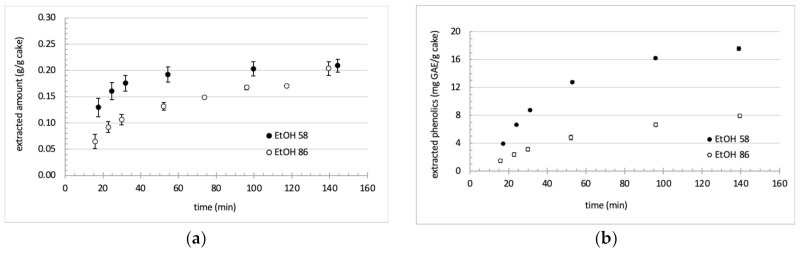
Extraction kinetics of (**a**) global extract and (**b**) total phenolic compounds using ethanol-based solvents.

**Figure 4:**
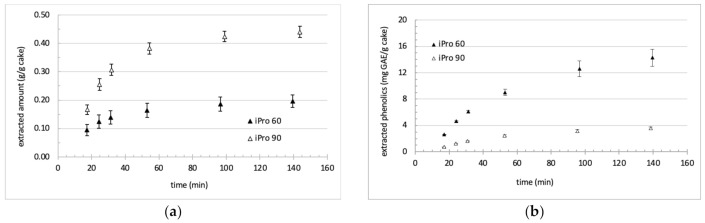
Extraction kinetics of (**a**) global extract and (**b**) total phenolic compounds using isopropanol-based solvents.

Although the final global yields were similar for EtOH 58, EtOH 86 and iPro 60 (Table 1), there was significant differences in the extraction kinetics (Figure 3 and Figure 4).

Looking first at ethanol-based solvents (Figure 3), the increase of the solvent hydration level increased the extraction rates of total extract and total phenols. The constant rate period that corresponds to the extraction of the most accessible compounds was about 30 min. In that period, extraction is limited by solubility, and obviously, solubilization capacities were higher for EtOH 58, indicating therefore that more polar species were extracted. After the constant period, extraction rates decreased because diffusion now contributed to mass transfer, and less accessible solutes were extracted. The extraction of polyphenols continued after 30 min, although for EtOH 58 the rate progressively decreased. For that solvent, the global extraction considerably slowed down after 60 min with a curve that almost reached a plateau. On the contrary, for EtOH 86 that was a less hydrated solvent, the extraction continued, indicating that the walnut cake still contained solutes to extract. The hydration level of the ethanolic mixture therefore influenced the extraction kinetics.

For isopropanol-based solvents, the water content also exerted a positive effect on the extraction of polyphenols. The effect was more pronounced than for ethanol-based solvents because of the very slow extraction rate observed with iPro 90. The extraction of polyphenols was not completed neither after 140 min, though the extraction rates started to decrease. The striking difference with ethanol mixtures comes from the global extraction. Isopropanol 90 led to a far higher yield compared with iPro 60, and the difference between the two yields was maintained till the end of extraction. For isopropanol mixtures, the largest part of extractible compounds was thus already extracted at 100 min (at the volume-to-solid ratio of about 20), whatever the water content of the solvent.

**Figure 5:**
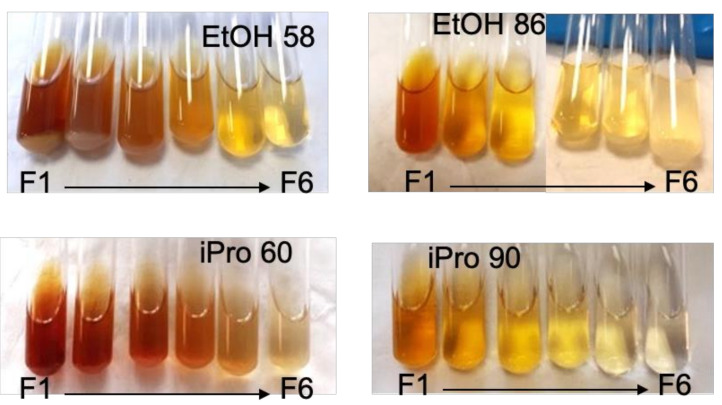
Visual aspect of fractions collected during extraction experiments, from the beginning of the extraction (F1) to its end (F6).

Data reported in Table 1 evidenced the fact that oil was extracted by all solvents, while kinetics curves showed that extracted amounts and polyphenols were distributed differently among the collected fractions depending on the solvents. The aspect of fractions is visualized in Figure 5. Looking first at the color, one can see that the color intensity was fading as extraction progressed and ranged from a reddish color in F1 EtOH 58 and iPro 60 to pale yellow, with even a colorless solution for F6. Samples produced by using a higher water content (alcohol 60 vs. alcohol 90) or ethanol-based solvents appeared as slightly more colored. Red to yellow colors may be associated with the presence of carotenoids and flavonoids whose multiple conjugated double bonds give rise to such colors. In the collected fractions, the highest concentration of polyphenols was obtained for F1 EtOH 58 (2.8 mg GAE/mL_solvent_) and F1 iPro 60 (1.9 mg GAE/mL_solvent_), which were the most colored samples, while the least colored fractions corresponded to phenols concentrations below 0.15 mg GAE/mL.

The presence of oil was visually detected as a separated phase or as dispersed droplets in the collected F1 and F2 samples, and up to F3 for iPro 90. A significant part of the extracted oil was therefore specially distributed over the three first fractions, that is, for a volume of solvent/mass of cake ratio of six. Some oil might exist in the other fractions, but at a concentration below the oil solubility limit. The solubility issue was also at stake for F1–F2 EtOH 58, in which a sedimented phase was noticed, and for F1–F2 iPro 60 but to a lesser extent. Fractions represent what was extracted at 60 °C but phase-separation or particles formation might occur when samples cooled down to room temperature because of the decreasing solubility with temperature. Though less visible on the pictures, fractions F4 to F6 EtOH 86 also contained a white bottom phase. This could be related to the specificity of EtOH 86 for which appreciable amounts of extracts were still produced after 80 min (Figure 3).

### 3.3. Self-Emulsification of Extracts

After desolventation by rotavap, the dried residues were rehydrated with pure water in order to assess their ability to self-emulsify. The hydration was carried out by adding water directly into the rotavap flask followed by sonication of the flask in an ultrasonic bath. Emulsions are created provided that there is an adequate balance between the oil and the surfactant molecules. Because of the different extracting ability of solvents and the different extraction kinetics, oil and polar surface-active molecules would be diversely distributed among the fractions whose concentrations were ranging from 120 mg_extract_*/*mL_water_ (F1 iPro 90) to 5 mg_extract_*/*mL_water_ (F6 EtOH 58).

**Figure 6:**
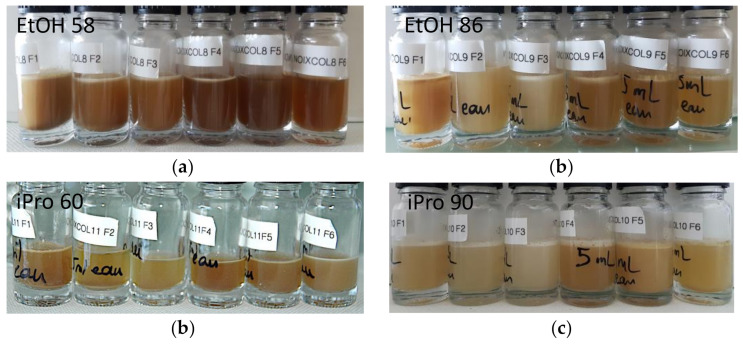
Aqueous dispersions obtained by hydrating the dried fractions collected during extraction with (**a**) EtOH 58, (**b**) EtOH 86, (**c**) iPro 60, (**d**) iPro 90.

The re-dispersion of extracts in water yielded to turbid and brownish dispersions (Figure 6).

**Figure 7:**
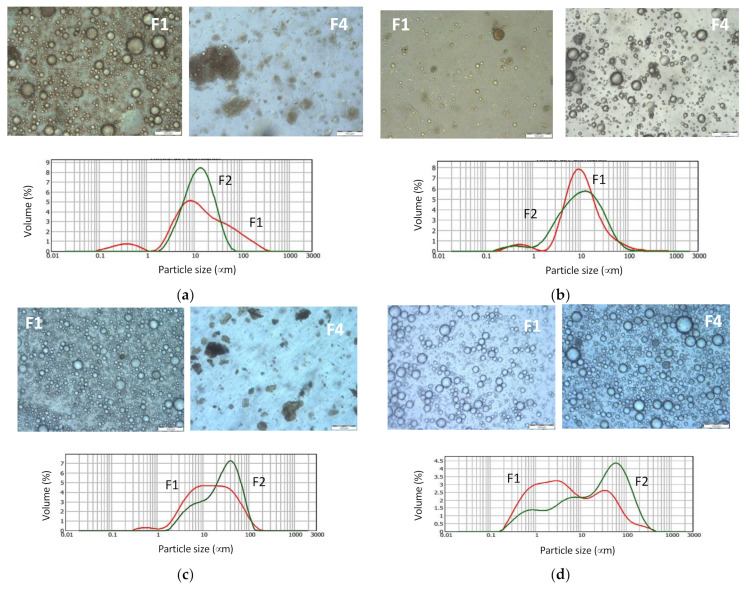
Aqueous dispersions obtained by hydrating dried fractions collected during extraction with (**a**) EtOH 58, (**b**) EtOH 86, (**c**) iPro 60, (**d**) iPro 90. Scale bar of 100 μm for microscopy images; image of F1 iPro 90 obtained after dilution of the aliquot.

For several samples, a thin creamy layer developed upon resting, therefore confirming the presence of an emulsion. The layer corresponding to a concentrated emulsion and creaming is a usual phenomenon caused by the difference of densities of the continuous and dispersed phases. The observation of the re-homogeneized samples by optical microscopy showed the presence of droplets of sizes up to 100 μm (Figure 7) proving therefore that endogenous surface-active components and oil were present simultaneously in several fractions. Since extracts were produced with alcohol-based solvents, the dispersion might contain compounds that became insoluble when the extracts were dispersed in pure water. Those materials are for instance seen in Figure 7 for the F4 EtOH 58 and F4 iPro 60 samples. It is worth noting that this particulated material can contribute to the creation of water–oil interfaces, as in the so-called Pickering emulsions [35].

Analysis by light scattering diffraction evidenced a polydisperse distribution as well, with sizes ranging from 0.1 to 300 μm in diameters (Figure 7). The analysis does not discriminate the nature of the diffracting objects; therefore, the reported distribution took into account droplets plus solid particles if present. Nevertheless, the sizes were rather fine if we consider the several steps of the dispersions fabrication (extraction-evaporation-rehydration) and the variations of solubilization that these steps could induce.

**Figure 8:**
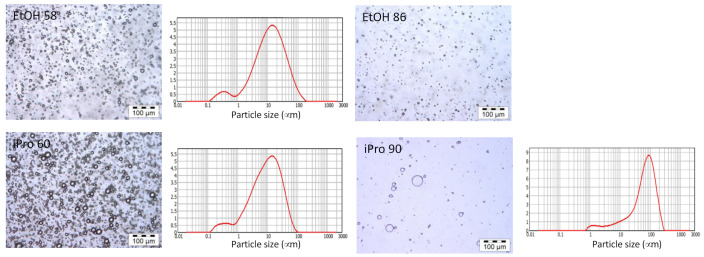
Characterization of washed F1 dispersions. Image of iPro 90 obtained after dilution of the aliquot; vertical axis for PSD is in volume %.

To access the size of the droplet population only, the F1 samples were washed and centrifuged to remove any solid particles [35]. Microscopy images and size distribution are given in Figure 8. No measurement is reported for EtOH 86 since the final washed sample was too dilute.

Microscopy images and light scattering analysis showed the presence of polydisperse droplets ranging from 0.1 to 100 μm, a range similar to that of the unwashed F1 samples. Compared to the EtOH 58 and iPro 60 samples, the distribution of the iPro 90 sample was shifted toward the larger sizes. It was shown previously that iPro 90 extracted a lot of oil compared to the other solvents. The larger emulsion size could indicate that the sample did not contain enough surface-active compounds to accommodate the large amount of oil in the form of small drops. For the other solvents, a better combination of less oil and enough surfactants was probably achieved, allowing for the formation of smaller droplets.

The strategy of combining the extraction of active molecules (such as oil, phospholipids, polyphenols and proteins) and their formulation in oil-in-water emulsions was recently tested by Delvar et al. [58]. Dealing with a high shear mixer or the twin-screw extrusion of passion fruits, the emulsifying ability of the filtrates was assessed. Droplets below 10 μm in diameter were obtained using an Ultraturax as an emulsifier. The filtrates, obtained after filtration on a 100 μm tissue, contained 8.7 to 17.2 g of dry matter/100 g_filtrate_ depending on the extraction process, among which 0.5 to 2.6 g*/*100 g_filtrate_ was of oil. In our work, the concentrations of unwashed F1 and F2 dispersions were 12.0 ± 1.2 g/100 mL_water_ and 6 ± 1 g/100 mL_water_ for the iPro 90 F1 and F2 samples, 8.6 ± 0.6 g/100 mL_water_ and 2 ± 1 g/100 mL_water_ for F1, F2 EtOH 58, 6 ± 1 g/100 mL_water_ and 2.1 ± 0.3 g/100 mL_water_ for F1, F2 EtOH 86 and iPro 60. The dispersions were simply obtained by adding 4 or 5 mL of water to the dried extract followed by 3 min in an ultrasonic bath and were not filtrated unless for specific purposes. No emulsification process more energetic than an ultrasonic bath has been applied.

To go beyond the proof of concept aimed at in this work, a scale-up of the extraction technique should be carried out. By producing fractions of higher masses whose composition could be analyzed, and by washing the aqueous dispersions in order to access the size of droplets population only, one can expect to get a deeper insight at the results interpretation. So far, we can however conclude that in terms of emulsifying potential, the less hydrated solvents iPro 90 and EtOH 86 provided a better balance of oil and surface-active molecules over the entire extraction process, since emulsions were seen in all fractions. On contrary, for the more hydrated solvents iPro 60 and EtOH 58, emulsification occurred in the two to three first fractions, while in the following fractions, more particles and few smaller droplets coexisted. The F3 and F4 fractions were usually more heterogeneous, and there were more variations between replicates.

## 4. Discussion and Conclusions

Environmental and sustainability concerns prompt increased interest in alternative solvents for oil extraction, by-product valorization and minimal processing. At the crossroads of these concepts, the work aims to look for potential solvents to combine the extraction of oil and phenolic compounds from a healthy oleaginous by-product.

More specifically, the study evaluated the performance of four hydro-alcoholic solvents of a quite-high hydration level (from 10 to 42 vol%) at producing complex extracts that would contain enough lipophilic and amphiphilic molecules to form emulsions upon rehydration and phenolic molecules for nutritional or antioxidative benefits. To refine the knowledge of the kinetics of extraction at short times and to optimize the consumption of solvent, a semi-continuous extraction design associated with a tight fractionated collection of the effluent was used. The results pointed out significant differences between the solvents.

Isopropanol 90 was the best solvent to recover oil from the press-cake. The oil extraction efficiency reached a value of 97% while the recovery stood in the range of 31% for EtOH 58 and iPro 60 and 36% for EtOH 86. Due to this efficiency, the total extracted amount for iPro 90 was larger than that obtained with the other solvents. The increase of the solvent hydration level negatively influenced the oil extraction yield but positively increased the recovery of the phenolic compounds for both alcohols. Ethanol was a better extracting solvent than isopropanol for this class of compounds, and the highest value of 17.6 mg GAE/g_cake_ was obtained by using EtOH 58. Regarding the self-emulsifying properties of extracts, several fractions contained enough surface-active material and oil to self-assemble as emulsions. These are generally the fractions obtained at short times, i.e., at a solvent/cake ratio of about 6–7. Emulsions can be also created by the presence of particles as in Pickering emulsions, so the presence of insoluble particles in the water dispersions should not be considered a drawback. Among the solvents, iPro 90 and EtOH 86 showed better performances in the sense that all extracts collected during the 140 min extraction were able to emulsify. Isopropanol 90 might be preferred because of the higher extraction yield it provides, but emulsions would be less rich in phenolic compounds. The most hydrated solvents were found to behave equally, with extraction yields in the same range for total extract, oil and phenolics and a limited emulsifying capacity of fractions.

From an industrial perspective, ethanol and isopropanol are suitable solvents to extract a complex mixture from a by-product such as walnut press-cake. On the basis of a 90% recovery of the extractable material, the use of iPro 90 and the collection of a single fraction cumulated up to a solvent volume-to-solid ratio of 11–13 is certainly the best choice, as it gives the highest mass of extract linked to the highest oil recovery. It can be assumed that the single fraction will emulsify, but this will have to be confirmed. If the target is the richness in polyphenols whilst oil is only a secondary benefit, one must choose EtOH 58 and a volume-to-solid ratio of 10 to obtain an extraction rate of 90%; but it will be at the expense of a lower extracted mass and an emulsification that may not be the best. Whatever the solvents chosen, it is worth recalling that alcohols will have to be separated from the extracts before their use as pure extracts or as emulsions.

## Figures and Tables

**Table 1:** Extraction yields of total extract (*n* = 3), total phenolic compounds (*n* = 2), oil (*n* = 3) and dielectric constants (Di) of solvents. Oil content of untreated walnut press-cake: 0.37 ± 0.01 g/g_cake_.

Solvent	Total Extract (g_extract/_g_cake_)	Total Phenolics (mg GAE/g_cake_)	Oil Extraction (g oil/g_cake_)	Di
EtOH 58	0.21 ± 0.01 ^a^	17.6 ± 0.3 ^a^	0.12 ± 0.03 ^a^	43.3
EtOH 86	0.20 ± 0.01 ^a^	7.9 ± 0.3 ^b^	0.14 ± 0.03 ^a^	28.9
iPro 60	0.20 ± 0.02 ^a^	14 ± 1 ^c^	0.12 ± 0.02 ^a^	38.3
iPro 90	0.44 ± 0.02 ^b^	3.6 ± 0.2 ^d^	0.36 ± 0.01 ^b^	20.7

^abcd^ Values with different superscript letters within a column are significantly different at *p* < 0.05.

## Data Availability

The datasets generated for this study are available on request to the corresponding author.
